# Ceritinib for *ALK*-Rearrangement–Positive Non–Small Cell Lung Cancer

**Published:** 2015-03-01

**Authors:** Nancy M. Nix, Kaitlyn S. Brown

**Affiliations:** St. Joseph’s/Candler Health System–South Carolina Cancer Specialists, Hilton Head, South Carolina; University of Georgia College of Pharmacy, Athens, Georgia

According to the American Cancer Society (ACS), lung cancer is the second most common diagnosis in the world and is the leading cause of death from malignancy in both men and women (ACS, 2014). Lung cancer accounts for 13% of new diagnoses and 27% of deaths from malignancy. The ACS (2014) estimated that in 2014, there were 224,210 new cases of lung cancer and 159,260 deaths. The death estimate is higher in lung cancer than in colon, prostate, and breast cancers combined (ACS, 2014).

Lung cancer is divided into two types: small cell and non–small cell. Non–small cell lung cancer (NSCLC) is the most common, accounting for 85% to 90% of all lung cancers (Goldenberg, 2014).

There are three primary histologic subtypes of NSCLC: squamous cell, adenocarcinoma, and large cell (ACS, 2014). Adenocarcinoma is the most common subtype. Research on adenocarcinomas has revealed several genetic mutations that have contributed to treatment response.

One mutation in particular is the rearrangement of the anaplastic lymphoma receptor tyrosine kinase (*ALK*) gene. The rearrangement forms a fusion gene between ALK and the echinoderm microtubule-associated protein-like 4 (EML4). The 5’ region of EML4 fuses to the 3’ region of ALK, creating the oncogene (Iwama, Okamoto, Harada, Takayama, & Nakanishi, 2014). The EML4-ALK fusion oncogene was first discovered in 2007 and is found to cause an increase in the growth of cancer cells (Gerber, Ghandi, & Costa, 2014; Goldenberg, 2014). Only about 2% to 7% of all NSCLCs have the ALK rearrangement (Goldenberg, 2014).

Currently, crizotinib (Xalkori) is the only other medication approved for the treatment of *ALK*-positive NSCLC. Crizotinib, an ALK tyrosine kinase inhibitor, was approved in 2011, after clinical trials showed superior efficacy with overall response and progression-free survival when compared with the standard NSCLC chemotherapy regimen (Gerber, Ghandi, & Costa, 2014). Despite the improvement short term with crizotinib, there is an issue with the development of resistance and relapse. The majority of patients relapse within 12 months, with only one-third of these patients relapsing due to a mutation to the ALK tyrosine kinase (Shaw et al., 2014). The remaining relapses result from several different mechanisms (Shaw et al., 2014).

The frequency of relapse prompted the research and development of alternative therapeutic options. Particularly, focus was placed on second-generation ALK tyrosine kinase inhibitors. In April 2014, the US Food and Drug Administration (FDA) approved ceritinib (Zykadia) for treatment of *ALK*-positive metastatic NSCLC in patients who have previously received or are intolerant to crizotinib (FDA, 2014).

## PHARMACOLOGY AND PHARMACOKINETICS

Ceritinib is an oral ATP-competitive ALK tyrosine kinase inhibitor. Its mechanism of action is similar to that of crizotinib, but ceritinib does not have any MET-binding properties (Shaw et al., 2014). Ceritinib does inhibit other receptors to a lesser extent, including insulin-like growth factor 1 receptor (IGF-1), insulin receptor, and ROS1 (Shaw et al., 2014; Marsilje et al., 2013). The structural differences contribute to the variations in binding and ALK affinity.

Ceritinib demonstrates dose-dependent growth inhibition and tumor regression in animal trials (Marsilje et al., 2013). Its average half-life is 40 hours, and steady state was achieved in 15 days (Shaw et al., 2014). When ceritinib is taken with food, its absorption is increased. The greatest impact comes with the consumption of meals that have a high fat content. It is primarily metabolized by CYP3A4 (Novartis Pharmaceuticals, 2014). Ceritinib also demonstrates modest 3A4 inhibition, so the concomitant use of other 3A4 substrates, inhibitors, and inducers is not recommended (Marsilje et al., 2013).

## KEY CLINICAL DATA

Currently, there is only one clinical trial with published data on ceritinib in *ALK*-rearranged NSCLC. In the phase I trial conducted by Shaw and colleagues (2014), the primary objective was to determine the maximum tolerated dose (MTD) for ceritinib. The MTD was defined as the dose associated with the highest probability that dose-limiting toxic events would occur in at least 16% but less than 33% of patients without being considered an overdose. Secondary objectives included characterizing the adverse effect and safety profile, pharmacokinetics, and antitumor activity.

Patients eligible for this study had locally advanced or metastatic cancer with the presence of the *ALK* rearrangement. The positive presence of the mutation was determined if at least 15% of tumor cells demonstrated the rearrangement using the fluorescent in situ hybridization (FISH) assay. Patients were eligible for this study regardless of previous treatment with crizotinib.

A total of 130 patients were enrolled: 122 with NSCLC and 8 with an alternative diagnosis. Eighty-three patients previously received and developed resistance to crizotinib. The median age was 53 years, and the majority of patients were Caucasian and female. All patients except one had an Eastern Cooperative Oncology Group (ECOG) performance status score of between 0 and 2. One patient consented to the study with an ECOG score of 2 but was changed to a score of 3 prior to starting the study.

The study consisted of two phases. The dose-escalation phase enrolled 59 patients. The expansion phase enrolled an additional 71 patients who received the MTD. During both cycles, daily dosing was continued in 21-day cycles until the disease progressed, the patient developed unacceptable levels of toxic effects, or consent was withdrawn.

The MTD was established at 750 mg. Among the 114 patients with NSCLC who received doses of 400 to 750 mg daily, the overall response rate was 58% (95% confidence interval [CI], 48%–67%). Of the 80 patients previously treated with crizotinib, the overall response rate was similar at 56% (95% CI, 41%–70%). The median progression-free survival was 7.0 months (95% CI, 5.6–9.5), 6.9 months (95% CI, 5.3–8.8) for those previously treated with crizotinib, and 10.4 months (95% CI, 4.6 to unable to estimate) in those who were treatment-naive.

Of those who had developed resistance to crizotinib, 19 underwent a tumor biopsy to further determine the mechanism of resistance. Of these patients, 12 did not demonstrate any additional genetic variations. The remaining seven patients did demonstrate some form of alteration, with five having secondary resistance mutations and the remaining two having gene amplification. However, during treatment with ceritinib, 7 of the 12 patients in the nonalteration group and 6 of the 7 patients in the alteration group showed a confirmed response. Therefore, the authors concluded that the reason for ceritinib activity after disease progression on crizotinib might be independent of the mechanism of resistance (Shaw et al., 2014).

The publication of the phase I trial included data only through August 2013. However, enrollment was continued through July 2013, and the additional results were used for FDA approval. A total of 304 patients were enrolled into the trial, with 163 presenting with *ALK*-positive NSCLC who had previously received crizotinib.

Both the overall response rate and the duration of response proved to be similar to the published results. The investigator assessment concluded an overall response rate of 54.6% and a median duration of response of 7.4 months. The Blind Independent Review Committee assessment concluded an overall response rate of 43.6% and a median duration of response of 7.1 months (Novartis Pharmaceuticals, 2014).

There are ten clinical trials currently recruiting patients to continue further analysis of ceritinib. However, only two studies are currently active. These phase II clinical trials (NCT01685060 and NCT01685138) aim to assess the overall response rate in patients with NSCLC and the *ALK* rearrangement, with one testing patients previously treated with crizotinib and the other testing treatment-naive patients (Novartis Pharmaceutics, 2012a, 2012b).

Future clinical trials aim to assess the use of ceritinib against standard NSCLC chemotherapy regiments in both untreated and previously crizotinib treated patients. Other future phase I and II clinical trials hope to evaluate its efficacy in those who have the *ROS1* genetic rearrangement and assess the pharmacokinetic parameters in patients with significant hepatic impairment.

## ADVERSE EFFECTS

In the published clinical trial information by Shaw and colleagues (2014), 66 of the 130 patients required at least one dose reduction. Eight patients permanently discontinued treatment due to an adverse event (Shaw et al., 2014). However, the additional data provided by the package insert demonstrated that 10% permanently discontinued treatment due to an adverse event, and 5% experienced fatal adverse drug events (Novartis Pharmaceuticals, 2014).

Of the 255 patients who received the 750-mg daily dose, the most frequent adverse effects reported included diarrhea (86%), nausea (80%), vomiting (60%), abdominal pain (54%), fatigue (52%), decreased appetite (34%), and constipation (29%). Other adverse events reported included rash, esophageal disorders, neuropathy, vision disorders, prolonged QT interval, and bradycardia. Several grade 3/4 toxicities per the Common Terminology Criteria for Adverse Events were reported, including diarrhea (6%), fatigue (5%), nausea (4%), and vomiting (4%).

In addition to these adverse effects, frequent laboratory abnormalities were also reported. They included elevations in alanine aminotransferase (ALT; 80%), aspartate aminotransferase (AST; 75%), creatinine (58%), glucose (49%), and lipase (28%); also reported were decreases in hemoglobin (84%) and phosphate (36%) levels. The most frequent grade 3/4 laboratory abnormalities were elevations in ALT (27%), AST (13%), and glucose (13%; Novartis Pharmaceuticals, 2014).

Based on the prevalence of adverse effects, several dose-limiting toxicities have been listed. They include diarrhea, vomiting, nausea, dehydration, hypophosphatemia, and elevation in ALT levels (Novartis Pharmaceuticals, 2014).

Four cases of interstitial lung disease were reported during the phase I trial (Shaw et al., 2014). All incidents were resolved with discontinuation of treatment and the administration of standard care. After Shaw and colleagues (2014) published the initial results, an additional six cases of pneumonitis were reported. Of the 255 patients, 3% experienced grade 3/4 pneumonitis. One case of pneumonitis was fatal (Novartis Pharmaceuticals, 2014).

Animal trials have shown embryo-fetal toxicity at dose equivalents of 750 mg daily. The toxicity led to delayed ossifications and skeletal variations. Therefore, use of ceritinib in pregnant women is not recommended. There are no safety data with ceritinib use in breast-feeding patients (Novartis Pharmaceuticals, 2014). 

## DOSING RECOMMENDATIONS

Ceritinib is available in 150-mg capsules. The recommended daily dose is 750 mg orally once daily. Novartis Oncology (2014) recommends the capsules be taken on an empty stomach. Patients should be advised not to consume food within 2 hours of administration of ceritinib.

Dose reductions from the initial daily dose occurred in 60% of patients. The median time to the first dose reduction was 7 weeks (Novartis Pharmaceuticals, 2014). Typical dose reductions are in 150-mg increments. Permanent treatment discontinuation is recommended for patients unable to tolerate 300-mg doses.

Other reasons for permanent treatment discontinuation include life-threatening bradycardia without concomitant medication, treatment-related interstitial lung disease or pneumonitis, QTc interval prolongation accompanied with additional cardiovascular symptoms, and ALT/AST levels greater than three times the upper limit of normal (ULN) along with bilirubin levels greater than two times the ULN. It is recommended that ceritinib should not be taken with other CYP3A4 inhibitors. However, if concomitant use of these agents cannot be avoided, a ceritinib dose reduction of one-third and rounded to the nearest 150 mg is recommended (Novartis Pharmaceuticals, 2014).

## IMPLICATIONS FOR THE ADVANCED PRACTITIONER

The determination of the presence of genetic mutations is imperative for choosing the correct treatment for patients with NSCLC. For patients with the *ALK* rearrangement, crizotinib is still the only medication approved for first-line treatment and should be initiated promptly. However, at any sign of disease progression, the practitioner should strongly consider changing to ceritinib.

Once a patient begins ceritinib treatment, liver function tests should be performed at least once a month and more frequently if elevations occur. Blood pressure, heart rate, electrocardiogram, electrolytes, and renal function tests should also be performed regularly. Patients should be educated on the signs and symptoms of hyperglycemia, pneumonitis, and gastrointestinal toxicities and should be encouraged to report any of them should they occur. In addition, patients should be educated on the importance of taking ceritinib on an empty stomach and not eating within 2 hours of its administration.

Like most oral oncolytics, ceritinib is a costly drug, estimated to approach $13,500 monthly (Goldenberg, 2014). However, Novartis currently offers a financial assistance program, which can be accessed at www.us.zykadia.com. Novartis may cover up to $36,000 per year in copayment costs for each eligible patient (Novartis Pharmaceuticals, 2014). This option should be considered for all patients before starting ceritinib.

## SUMMARY

Ceritinib is the only medication indicated for *ALK*-rearranged NSCLC in patients whose disease has progressed or who are intolerant to crizotinib. Clinical trials show efficacy in both crizotinib treatment-naive and previously treated patients. However, its use does present several significant toxicities, which have led to fatalities. Further clinical trials are needed to assess its full safety profile and to determine whether ceritinib may be an appropriate agent for treatment in additional settings.
